# Cavity Preparation for Porcelain Fillings

**Published:** 1904-11-15

**Authors:** W. T. Reeves


### CAVITY PREPARATION FOR PORCELAIN FILLINGS.

BY W. T. REEVES, D.D.S.

In presenting cavity preparation for porcelain inlays, we are presenting a new line of work that has been very little written on, and, I think, largely for the reason that it is hard to convey any adequate conception of the work without it being profusely illustrated, or with the aid of models.

The use of porcelain for fillings entails cavity preparation entirely different from anything heretofore used. The orifice of the cavity has got to be larger than the interior, so that whatever form of matrix is used and burnished into the cavity can be withdrawn. There are a great many other features that are exclusive essentially to porcelain fillings.

I shall give seven conditions that should be followed in every capacity.

First of all, *remove all decay.* This should be essential in all classes of work. There are a very few places where it might be permissible to leave a little of the decay; but, for a general principle, all decay should be removed.

Second, *extend margins to glistening enamel.* In the use of porcelain we have a condition that is different from any other material. The surface of the porcelain brings protection to the adjoining tooth surface, and consequently it is not necessary to make extensions for prevention, on the principle that we do it when we employ gold amalgams and cement as the filling material. A good enamel margin should be cut until you reach glistening enamel. This should be determined by the operator, that is how much cutting should be done in the filling of the tooth; the extension of the margin is sufficient to give all that is necessary in that connection, as compared with the extension for prevention.

*Divergence from parallel lines.* A great many at present, in writing on the subject of porcelain for filling, are advocating the formation of cavities with parallel walls. In one way it would add something in the matter of retention, but it complicates in several ways, so that we lose more than we gain.

A cavity that has parallel walls would necessitate, for accurate fitting, to work from an impression and form the matrix on the outside of an impression, so that whatever was baked into the matrix would be as large as the cavity. One of the points that is made in connection with burnishing direct into the cavity is that the matrix would be eliminated.

Whatever is baked into that cavity, or whatever is burnished into that cavity, when the matrix is stripped off, will lack in diameter the thickness of the material on both sides; in depth it would fill the cavity, because the material extends over the surface of the tooth to the same extent that it takes up at the bottom of the cavity, so that in depth it would fill the cavity, but in diameter it loses on both sides; it would lack the thickness of the material. If we just reach over the parallel walls, we have a cavity that the material that is burnished in them will leave an orifice as wide as that in the cavity. When the porcelain is baked into the matrix, it should set into the cavity as a wedge would set into any place, and in that way practically eliminate the thickness of the matrix.

*The undercuts should be obliterated,* because if we had undercuts it would prevent the withdrawal of the matrix without distorting it or warping it, so that whatever was baked into the matrix would not fit the cavity. We go to another of the models and we will speak of the different ways that such undercuts can be obliterated—margins beveled to a knife-edge with the outer surface of the teeth. If a joiner were making a joint of a plank, or any piece of timber, and it was sawed square, then the joint would be what I would call a butt-end joint; but if I wanted to make a very close joint, I would saw that so it would make a lap-joint, and in that way I could make a joint that would have a great deal less space than one that had a butt end. Now we want to do the same in connection with preparing our cavities, so that when the inlay goes into the cavity it goes in on the principle of a lap-joint instead of a butt-end joint. In that connection I have three diagrams, this representing one tooth and this another, copied the best I could from articles that appeared within this year in the magazines. In both cases they represent what would be a butt-end joint, this representing here the thickness of the enamel, and then the pressing into the cavity, so that when it is fitted in there, we have a square seat at the top and in both places it would amount to a butt-end joint. This is a flat surface, and that at right angles to it, making both of these what would be a butt-end joint. In this lower one I have tried to represent the formation, whatever formation we want to give it, as far as the formation of the labial surface is concerned.

But in looking at that cavity from the other view we would have the margins sloping in from the outer surface of the tooth, and in that way we would have what would be the same as a lap-joint in the illustration, as a joiner would make a joint.

*Seat formation for frictional retention.* All cavities should have seat formation for frictional retention, and that would be what is termed a saucer-shape in the cavity, and would then give seat formation for frictional retention.

*Seat formation for setting purposes.* An inlay to be in its place, if it were in a strictly saucer-shaped cavity, when the cement is put into the cavity, it is very easy to slide a little out of place; one margin may be very slightly depressed, another one will be correspondingly raised, and in that case all portions of your inlay are just a little changed out of adaptation to what the matrix was, part of it would set and part of it would be out of position. Now, some formation in the cavity that will very nearly insure the inlay going absolutely to place will bring every part of it into its proper position and give us the adaptation which,

I believe, is the secret of the success of their retention.

We will start with a cavity on the labial surface, and when that would occur on all the teeth at contact point—that is, contact point cavities in any of the teeth, anterior or posterior teeth cavities, which in their inception are very simple, and a No. 3 or 4 round bur would remove all the decay and carry us to good, sound tooth tissue——we would have a strictly saucer-shaped cavity.

Now we wish to avoid that for the purpose of retentive form and also for the purpose of its being set in its proper position. To do that the use of the small bur around the floor, run around the cavity, would flatten that floor, and it would be but a very little cutting that it would have to do; it would flatten the floor. In doing that flattening, it is well to do it in a form that will make an identifying form of inlay, because while we have a matrix on an inlay of this nature, oval or round, it is easily identified—our matrix gives us the shape of the teeth, gives us the same impression, the shape of the tooth; but when the matrix is stripped off, there are a great many of them that you could not tell which was which, and they are near enough alike as to be hard to detect when you have to set them into the teeth; but if, in flattening the floor for frictional retention form and also for form for setting purposes, you will make one end a little wider than the other, then the inlay can go only into the place or the position into which it was burnished. If it is of a nature which is hard to identify as to color, you have made no variation in the color which will identify, which would be that at the cervical portion, toward the cutting edge; after you have stripped the matrix off and put it in the cavity previous to putting the cement in, take your pen and with a spot of ink on the outer surface of the inlay, far enough away from the margin that the ink will not run underneath and remain on the inside of your inlay, you can then set the marked inlay on the table, and when you have come to set it you can pick it up with an absolute certainty that you know into which position it should go, without looking to the inside to detect the little difference in its surface.

In regard to the finishing of the margins to a knife-edge, one of the best ways that you can determine that is to do so by the position your bur or stone is occupying in doing the cutting. If the bur or stone is cutting with the diameter of the bur at the margin, then the slope has got to be so that they will make no undercut; but if you are cutting so that the diameter was below the margin, the chances are you might get a little undercut, and there are lots of positions in the mouth that it would be hard to detect that little difference.

Next to that class of cavities would come those in the contact point that have developed to a little larger extent, particularly in the anterior teeth, developed, possibly, to the extent that this cavity on this side illustrates. Some of them may have been filled with gold fillings that require refilling, that make cavities this size larger. Remove the decay and come to the glistening enamel, and then, in the seat formation of such a cavity, make it of a triangular formation the base of the triangle gumwards.

Now those cavities that occur in the approximal surface, that have extended to the point of encroaching upon or weakening the corner of the tooth, or incisal edge, to the extent that if you were to fill that cavity with gold it would be necessary to cut it off. If there is any dentin remaining between the plates of enamel, it is perfectly safe to leave a corner of that kind and fill it with an inlay, because the inlay will strengthen that tooth to the strength of the tooth previous to any decay encroaching upon it. If decay has removed a portion or all of the dentine remaining between the plates of enamel, then it is up to the individual, at the time, to decide whether such plates filled in with a little cement will still leave them strong enough. In the majority of cases they will be strong enough. But if you have dentine between these plates, it is perfectly safe then to fill such cavities with an inlay; but if you were filling with any other material, it would be necessary that you cut off the corner and make a cutting edge cavity. Now I have been asked, "Why not cut the corner right down as you do for a gold filling?" The reason would be this: that it is a small cavity to fill when you have a corner remaining, but if you are restoring the corner, then you have a contour to build out that takes more time, and you will make the filling in much less time and will be perfectly safe. Now that will apply particularly to laterals where the corner runs off diagonally in the majority of cases when it is encroaching upon the cutting edge of the tooth. Next to this would come cavities that involve more or less of the cutting edge. In all this class of cavities, the floor at the cervical margin, you would at least cut it so that it would be parallel with the line of the cutting edge, giving that much of a shoulder at the cervical portion. In the majority of cases your cavity will shape up so that you will have more than that. The only other feature in connection with these cavities, on general principles, is the preparation of the cutting edge. Instead of making the cutting edge flat, as a stone or disc would cut it, or as you could cut with the Arkansas stone, which would cut it round to about a quarter of the distance from the cutting edge to the gum; then from that point make a triangular formation for setting purposes. An inlay made for such a cavity, the more it is wedged the tighter it will go to its correct position, and better the result.

In lots of cases that have gold fillings, where the filling has been cut across the cutting edge for anchorage, the labial plate of the enamel will frequently break. If it was to be refilled with gold, we would from necessity have to cut a straight or a perfectly-curved line, and remove the enamel and dentine at this portion of the tooth. If we were to fill with the porcelain and cut in that manner, we would have, at this point, a very attenuated portion of porcelain; it would be very brittle, and in the fusing of the inlay would undoubtedly break at that portion. For the purpose of getting the strength of the porcelain, we would have to cut away more.

In the matching of colors, which we do when we match porcelain to the teeth, we can take advantage of the fact that a curved line is deceiving to the eye, and a joint would be much less conspicuous than a straight line, and we can join right into the lines of the cavity on two curves. Join to hold the curve or make the old curve true again. Take advantage of the fact that a curved line is deceiving to the eye. We also have the additional advantage of the shoulder formation, the frictional retention throughout, and in a great many cases, where it is not broken, but where we can leave a curved line in the formation, it is being followed very largely on account of the chance of its deceiving the eye. In connection with that, if the cavity is of peculiar character, and that is not very frequent, a depression is cut at the point that the matrix is burnished into, and then the resultant baking will leave a little bit of porcelain to fill that depression, will give a great deal stronger retention than any pins that you can bake into the cutting edge of inlays and have the corresponding hole cut into the tooth for such pins to set into.

In taking up the cavities occurring in molars and bicuspids, practically one class of description will cover all cases of that kind, great and small. In a cavity that is directly at the contact point, and as they occur in the occlusal surface of molars and bicuspids, the decay will progress either labially or lingually in one direction. Now, instead of being necessary to cut at the opposite side to the extent you would in filling with other materials, you can shape up your cavity as it has naturally decayed.

A cavity which is broader at the cervical margin than it is at the occlusal corners is a cavity that has been frequently cited as being impossible to fill with porcelain, whereas you can easily tuck the gold around at these corners and fill with gold. Now it is only a matter of just a little more separation for that class of cavity, and the separation can be accomplished with the use of separators, afterward packing gutta-percha and allowing the patient to go for a few weeks, and then an inlay can be made which will pass up in the space and even set into the cavity. That is where we have the extremely-heavy corners, the occlusal corners, and we do not wish to cut them away. If there were any undermining of the occlusal margins of the bicuspids by decay, however slight, cut the corners away and shape it in a manner to have a cavity that the matrix would withdraw upward, and practically require no inter-proximal space. If your filling can be made to go in there and you need no space for finishing, and if the opposite side has not been involved and no probability of its being so, yet there is a small fissure decayed, I would cut the fissure out and fill with a simple gold filling. It can be cut with a No. 2 or 3 size round bur and filled with a gold filling very quickly; then shape up the cavity on the approximal surfaces as though there were nothing in connection with the sulci of the tooth to consider at all; but if the pit opposite is involved and decayed, or decay has started, then cut it out completely and make the inlay to carry across and fill the opposite pit.

If there are two inlays on the approximal surface of bicuspids, you carry the inlay far enough so that when you shape up the opposite cavity, you can cut against your inlay and shape the second cavity as though that were all there was to consider in connection with that tooth, making the joint in the center, against the inlay, the same as you would against the tooth.

In connection with the cavities at the cervical portion of the molars or bicuspids, or in the anterior teeth, frequently the cavity will extend beyond the enamel; we will cut and reach good glistening enamel at this point, but the dentine is decayed beyond. If we were to fill this with gold fillings, it would be necessary to cut so that we had no thin plate of enamel, because the packing of the gold in such a cavity would very likely break that plate of enamel away; but for inlays, when you have reached good glistening enamel, so that you have good tooth substance, then you could fill in behind that with a little cement and shape your cavity, just as though it were good dentine that you were working with in the inlay setting there, and you would not endanger that part of the tooth, but make it perfectly safe.

One other class of cavities—that class occurring on the labial surfaces, where the most of the cavity is upon the root portion of the tooth, where in the majority of cases it is considered necessary to pack such a cavity with gutta-percha in order to force the gum out of the way. In handling that class of cavities, I do not pack to force the gum tissue out of the way, because in most all cases gum tissue packed away from a cavity in that manner does not come down and cover over the filling as it should; but, in order to avoid the hurting of the gum, such cavities can be made with a small gem-stone that is used for cavity cutting. You can use that and obviate the coming of the diameter up to the margin, because then you would lacerate the gum, but cutting into the cavity you can remove all decay and get margins that are sufficiently good at these points. That kind of cutting will result in an undercut which will not do any harm, for the reason that the inlay will start from this end and reach up into the undercut and down into the cavity. The burnishing of the matrix for that class of cavity can be accomplished very nicely by burnishing from this end, simply forcing the gum gradually back by pressure ahead of the platinum, numbing the gum as you push up, and in that way accomplish it with comparatively little pain to the patient.—*Western Journal.*

